# Surgical management of an infected external iliac artery interposition graft with a bioengineered human acellular vessel

**DOI:** 10.1016/j.jvscit.2021.10.002

**Published:** 2021-10-14

**Authors:** Christy Guth, Thomas Naslund

**Affiliations:** Department of Surgery, Vanderbilt University Medical Center, Nashville, Tenn

**Keywords:** Bioengineered human acellular vessel, Biologic graft, Infected vascular graft, Vascular bypass

## Abstract

Infection of prosthetic vascular grafts can manifest as pain, pseudoaneurysms, or arterial insufficiency in the leg. We present the case of a female patient with a medical history of a right external iliac artery endofibrosis, with a persistently infected synthetic iliofemoral bypass graft, which we replaced with a bioengineered human acellular vessel. At the 12-month follow-up visit, the clinical and radiologic studies demonstrated adequate human acellular vessel patency, with no signs of infection, stenosis, or pseudoaneurysm. Subsequent to the initiation of hormone therapy and cessation of antiplatelet therapy, the patient developed graft thrombosis. She continued to do well after restoration of patency with lytic therapy. At 22 months, secondary patency has been maintained with continued anticoagulation therapy, and the patient has remained asymptomatic.

Prosthetic vascular graft infections are a challenging complication with an incidence of 0.5% to 6% after surgical bypass.^1^ Prosthetic vascular graft infections are often treated with prosthetic graft excision and replacement with more infection-resistant biologic material. Historically, autogenous vein or cryopreserved cadaveric allograft has been used instead of extra-anatomic bypass with prosthetic material outside the infected wound.^2^^,^^3^ Recently, the use of a bioengineered human acellular vessel (HAV) has been documented to have low infections rates. However, to the best of our knowledge, its use to replace an infected synthetic graft has not yet been reported.^4^ In the present report, we have described the case a 42-year-old athletic woman with a history of an infected synthetic graft of the right external iliac artery, which was subsequently replaced with a HAV, in a U.S. Food and Drug Administration–approved expanded access case. Our patient provided written informed consent for the report of her case details and imaging studies.

## Case report

The patient was a 42-year-old white female cyclist and lifelong nonsmoker. She had presented with perigraft fluid collection and presumed infection of a right iliofemoral Dacron bypass graft and ipsilateral claudication. The patient’s medical history was significant for external iliac artery endofibrosis that had been bypassed 4 years previously at an outside hospital using an 8-mm Dacron interposition graft from the proximal external iliac artery to the common femoral artery.^5^, ^6^, ^7^ The bypass was complicated by stenosis at the inguinal ligament and graft infection. Initial antibiotic management was dictated by outside hospital providers before her referral to our practice. Graft excision and replacement were recommended. However, the patient did not have an adequate saphenous or superficial arm vein and, as an active athlete, requested alternatives to deep femoral vein or arm vein harvest owing to the risk of associated morbidity and potential swelling. Cryopreserved tissue was considered unfavorable given the poor longevity in a young patient. Extra-anatomic grafts can be used to provide bypass outside the infected field and have reasonable patency of 80% at 5 years.^8^ In an active cyclist, however, the risk of kinking is increased by the repetitive hip flexion. Thus, we thought it best to avoid an anastomosis in an unaffected limb. Therefore, the use of the HAV was requested from and approved by the U.S. Food and Drug Administration within 48 hours under an individual patient expanded access investigational new drug application (investigational new drug no. 18891).

**Fig 1:**
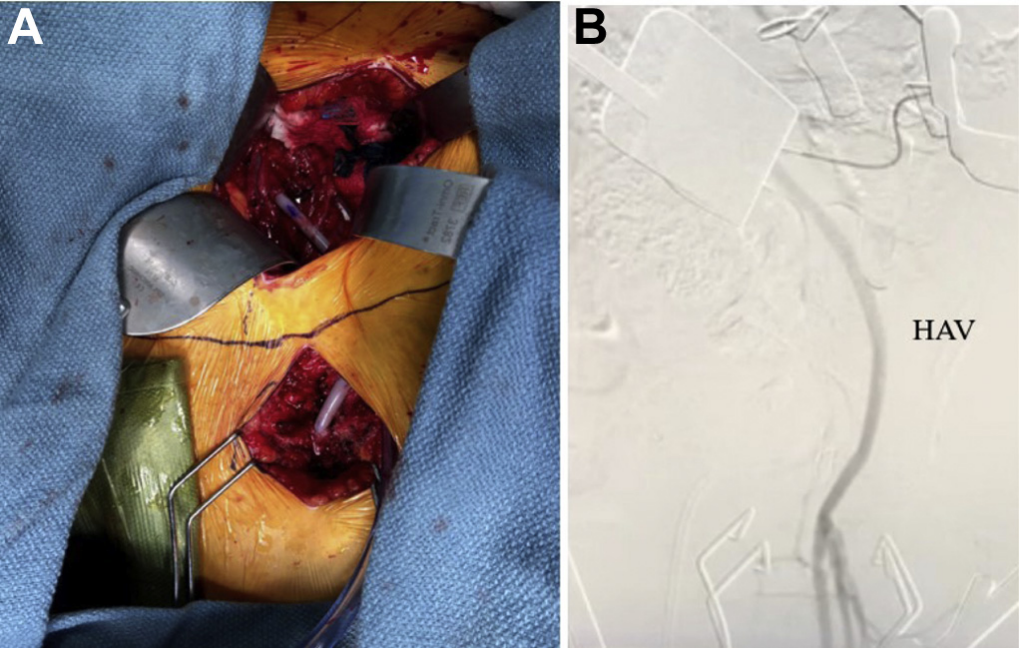
**A,** Incision in the right lower quadrant exposing the implanted human acellular vessel (HAV), anastomosed proximally at the common iliac artery, tunneled lateral to the native artery, under the inguinal ligament, and anastomosed distally to the common femoral artery. **B,** Arteriography of the implanted HAV demonstrating adequate perfusion from the proximal anastomosis at the common iliac artery to the distal anastomosis at the common femoral artery.

HAV implantation was performed through the patient’s previous incisions above and below the inguinal ligament. After exposure and control of the common iliac artery and common femoral artery, a tunnel was created lateral to the prior graft and running under the inguinal ligament for placement of the HAV. The Dacron graft was completely excised and the native artery debrided to healthy tissue. A 6-mm HAV was chosen and anastomosed end-to-end using 5-0 Prolene suture (Ethicon, Raritan, NJ). After blood flow was restored, patency of the vascular reconstruction with the HAV was confirmed by intraoperative completion angiography, with the needle placed in the common iliac artery. Digital subtraction arteriography was performed in two planes with contrast enhancement and demonstrated wide patency of the graft and anastomoses proximally and distally (Fig 1).

The patient recovered well from surgery and was discharged on postoperative day 4. Intraoperative tissue cultures were negative for bacterial growth. Under the advisement of the infectious disease specialists, the patient received empiric antibiotic treatment (vancomycin and ceftriaxone) for 6 weeks and was then transitioned to oral doxycycline for an additional 6 months. She also started daily aspirin 81 mg.

**Fig 2:**
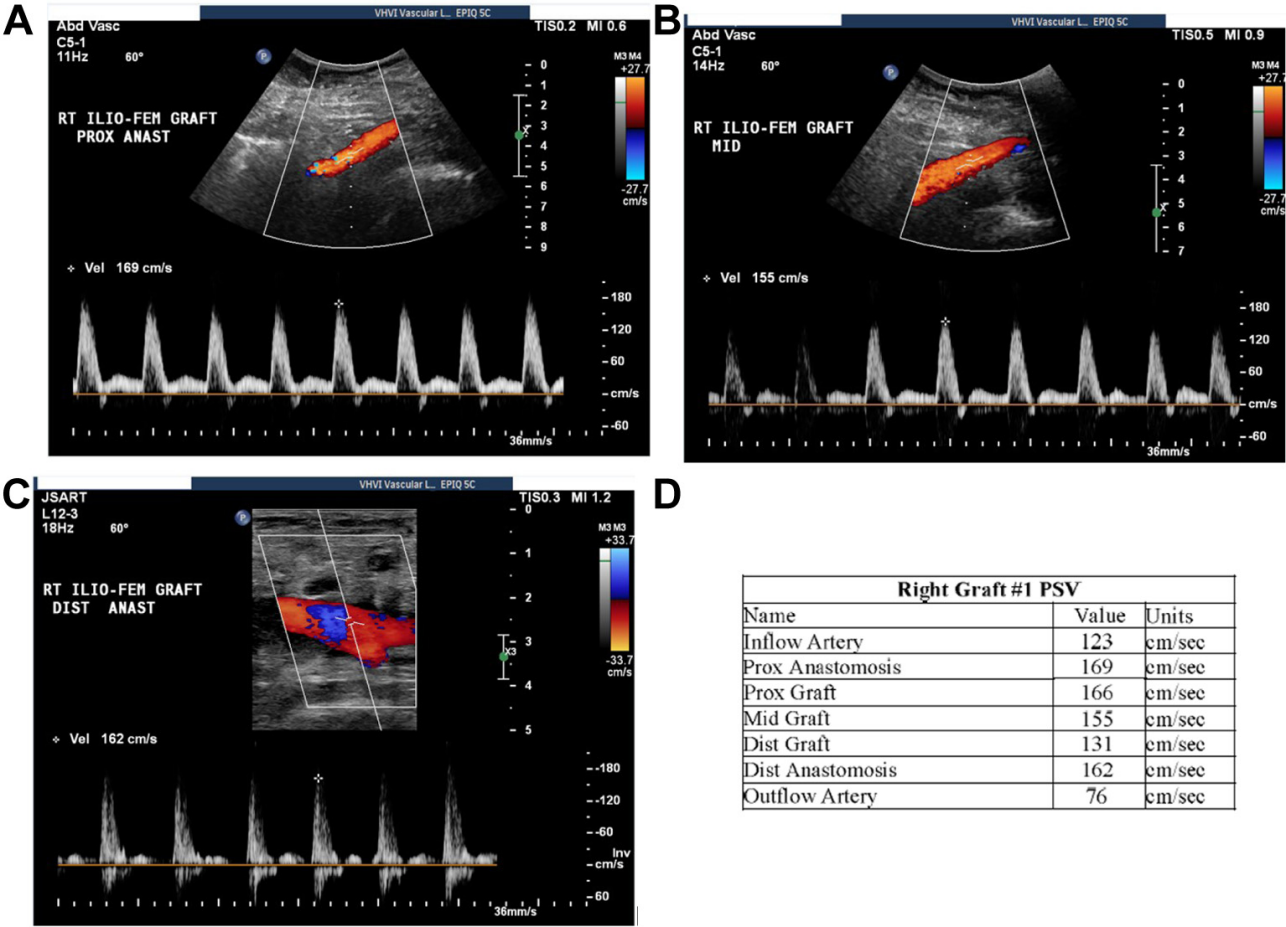
Selected duplex ultrasound images at 1 month after human acellular vessel (HAV) implantation for the proximal anastomosis (**A**), midgraft (**B**), and distal anastomosis (**C**). **D,** Peak systolic velocity (*PSV*) values for the right graft. *Anast,* Anastomosis; *Dist,* distal; *Ilio-Fem,* iliofemoral; *Prox,* proximal; *Rt,* right.

**Fig 3:**
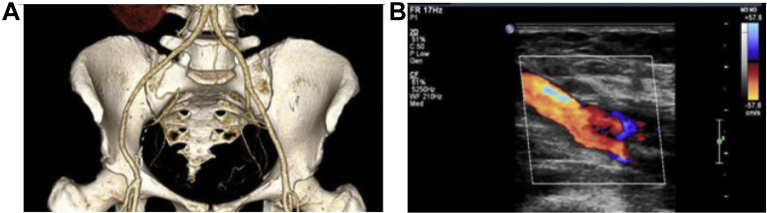
**A,** Three-dimensional reconstruction of computed tomography angiogram at 1 year of the proximal anastomosis of the human acellular vessel (HAV) and distal anastomoses at the common iliac artery to the right common femoral artery. **B,** Duplex ultrasound color flow image at 1 year of the HAV at the proximal anastomosis, midgraft, and distal anastomosis.

The patient underwent follow-up examinations at 1, 3, 6, 9, and 12 months postoperatively (Fig 2). At 1 month, duplex ultrasound revealed a widely patent graft without evidence of perigraft fluid. In addition, at the 1-month examination, the patient was active and walking, with minimal claudication, which had improved compared with the preoperative assessments. At 3 months, the follow-up imaging findings indicated that the HAV appeared to have the same diameter as the adjacent artery and the patient continued with oral antibiotic therapy. At 6 months, computed tomography angiography confirmed the HAV to be widely patent with no evidence of perigraft fluid or infection. Noninvasive physiologic studies of the lower extremity arteries were conducted at rest and during exercise. The ankle brachial indexes (ABI) were normal, and antibiotic therapy was discontinued. The patient experienced minimal-to-mild thigh claudication during exercise but did not report below-the-knee pain when walking. However, at 9 months, a repeat of the noninvasive physiologic studies at rest and during exercise showed that the ABI had decreased slightly to 0.73 from an ABI of 1.05 at rest. Duplex ultrasound and computed tomography angiography of the lower extremity arteries (Fig 3) showed a patent HAV, with no signs of stenosis, infection, or pseudoaneurysm. At 12 months, the follow-up examination findings and laboratory test results were unremarkable for HAV-related complications or infection. It is unclear why her exercise ABI had previously decreased; however, at 12 months, the ABI had returned to 1.11 to 1.17.

At approximately 16 months postoperatively, the patient had developed progressive menometrorrhagia, leading to cessation of aspirin and initiation of hormonal therapy. However, at 18 months, she had presented with thrombosis of the HAV and right common iliac artery. Uterine embolization with subsequent initiation of transcatheter thrombolysis of the common iliac artery and HAV restored normal flow. No technical concerns were identified with the graft. The patient has received oral anticoagulation therapy after recovery from her hysterectomy and was doing well at 22 months postoperatively.

## Discussion

When considering the materials to use for replacement of an infected graft, the most widely used choices have been autologous, cryopreserved, and prosthetic grafts.^1^^,^^9^^,^^10^ To the best of our knowledge, the use of a novel bioengineered HAV as a bypass graft for treatment of an infected prosthetic graft has never been studied. Multiple studies have demonstrated that the HAV, as a blood vessel that is cultured in vitro and then decellularized to generate a conduit that contains no living cells, preserves its mechanical properties, similar to native human vascular tissue.^11^, ^12^, ^13^, ^14^, ^15^ Once implanted, the vessel repopulates and remodels with the host’s own cells, turning the HAV into living tissue, with early evidence demonstrating a low risk of infection after implantation.^16^, ^17^, ^18^

The present report is an important early description of the use of a human tissue engineered vessel in a known infected field, in which a previously placed synthetic graft had become infected. Because of this infection, the lack of autologous graft options, and the risks associated with revascularization with another synthetic graft, the HAV was used as an alternative treatment option for our patient. Our patient was found to have had a culture-negative infection. Thus, the performance of a HAV graft in a culture-positive field, with more virulent bacteria, is unknown.

At 22 months after implantation of the HAV, the patient had required one intervention to restore graft patency after thrombosis. This could have resulted from the initiation of hormone therapy or cessation of antiplatelet therapy in the setting of incompletely endothelialized tissue. Although nearly complete endothelial coverage has been confirmed in HAVs as early as 44 weeks, additional data regarding the risk of HAV thrombosis to guide the type and duration of antiplatelet or anticoagulant therapy is necessary when considering the risks of this approach.^11^ A hematology consultation resulted in initiation of anticoagulation therapy. She has continued to demonstrate significant clinical improvement, including resumption of her regular physical activity.

## Conclusions

The present case has demonstrated how a bioengineered HAV can be used as an arterial bypass conduit in the setting of prior graft infection. This could provide a unique alternative to other graft options for the treatment of infected vascular prostheses. Further studies to demonstrate the utility of this approach are recommended.
